# Healthcare providers’ perspectives on sustaining respectful maternity care appreciated by mothers in five hospitals of Rwanda

**DOI:** 10.1186/s12912-024-02017-5

**Published:** 2024-06-28

**Authors:** Alice Muhayimana, Irene Kearns

**Affiliations:** 1 Department of Nursing Education, School of Therapeutic Sciences, Faculty of Health Sciences, University of Witwatersrand, Johannesburg, South Africa; 2 School of Nursing and Midwifery, University of Rwanda, Kigali, Rwanda

**Keywords:** Positive childbirth, Appreciative care, Best practices, Mother-friendly care, Rwanda

## Abstract

**Background:**

Childbirth reserves respect, as emphasized by the World Health Organization in 2018, and the focus towards the need for positive, dignified delivery experiences has become an integral aspect of Respectful Maternity Care (RMC). It is a known fact that RMC is pivotal for favourable birth outcomes and contributes to the satisfaction of maternity care. The absence of RMC negatively affects women's and newborns’ rights. The study aimed to explore healthcare providers’ perspectives on sustaining RMC actions that mothers previously reported.

**Methodology:**

This study was conducted in five hospitals in the Eastern province of Rwanda, involving 5 Focus Group Discussions (FGDs) with midwives and nurses. For interviews, we purposively selected 5-unit managers and five physicians. Additionally, 40 midwives and nurses were recruited for the FGDs. The research utilized the Dream phase of Appreciative Inquiry (AI) for interviews and Focus Groups. Data collection aimed to gain insights into Healthcare Providers’ perceptions of how RMC is provided and how to establish and sustain RMC in Rwandan health facilities. Nvivo 12 was employed for organizing codes and creating a codebook, and thematic analysis was applied.

**Results:**

Four themes with sub-themes emerged. Namely, 1) **Women-centered care,** with Compassionate care, Privacy and confidentiality maintenance, Information provision and Liberty in decision making, Effective communication, Family involvement, Cleanliness, and Equality care. 2) **Professionalism compliance** with Motivated staff, Teamwork, Continuous development, Quality work provision, and Community trust. 3) **RMC encounters** 4) **RMC sustenance.**

**Conclusion and recommendations:**

The continuous pursuit of high RMC standards in Rwanda involves improving childbirth experiences through utilizing existing resources, ongoing improvement, and sustaining achievements. Key recommended actions in this study for sustaining RMC encompass promoting women-centred care, enhancing healthcare provider attitudes, ensuring professionalism, building community trust, maintaining conducive health facility environments, and involving leadership.

**Supplementary Information:**

The online version contains supplementary material available at 10.1186/s12912-024-02017-5.

## Introduction

In the era of striving to achieve the Sustainable Development Goals (SDGs), countries worldwide, including Rwanda, aim to meet all goals and targets by 2030, employing various strategies. This study specifically focuses on one target of SDG number 3: to end all preventable deaths of maternal and newborns [1], and one target of SDG number 5: to eliminate all forms of violence against women and girls [2].

Childbirth, a significant human experience, deserves respect. The intrapartum and immediate postpartum period poses a high risk for both mother and newborn, necessitating high-quality care [3]. White Ribbon Alliance (WRA) defined Respectful maternity care (RMC) as a human right of every woman and her baby to be treated with care, respect and dignity, free from harm, and to maintain absolute liberty and autonomy [4]. Therefore, RMC is not an option; it is a basic human right. World Health Organization (WHO) in 2018 stated that RMC extends beyond the mere prevention of maternal morbidity and death. It emphasizes the need to ensure that women have a pleasant delivery experience, which entails being treated with utmost respect and dignity [5, 6]. In 2018, WHO shifted its focus towards ensuring a positive childbirth experience, emphasizing its importance [7].

Mistreatment towards mothers during childbirth contributes to the poor quality of maternity care they receive [8]. Studies argue that Disrespect and Abuse (D&A) can inflict long-term psychological damage and emotional trauma, diminishing women’s confidence and self-esteem [9]. Being disrespected may lead to feelings of shame, sorrow, insecurity, distrust in healthcare staff, a sense of powerlessness, and reluctance to seek help [9]. Memories of labour and delivery experiences persist throughout a woman’s lifetime and are often shared with others [8, 10]. Disrespectful and abusive care instils fear of future utilization of maternity services, reducing their use, which, in turn, hinders safe motherhood and contributes to increased adverse maternal and neonatal outcomes [8, 9]. D&A is a violation of fundamental human rights [4, 7].

Rwanda has made significant strides in maternal and neonatal health, achieving Millennium Development Goals through initiatives like increasing skilled birth attendants, family planning, and Community Health Workers (CHWs) since 2009. CHWs play a pivotal role in promoting maternal and newborn care at the household level. The introduction of RapidSMS technology in 2010 synchronized health systems and increased ambulance services, improving the transfer of pregnant women to health facilities. Mandatory health insurance coverage since 2008, with 91% coverage, ensures access to healthcare [11].

Rwanda’s notable progress in prioritizing women’s rights has indeed made significant strides in promoting gender equality, particularly in political representation. The country has surpassed many others regarding the percentage of women in parliamentary positions, showcasing a commitment to empowering women at various levels of society [12]. Ultimately, this commitment to promote women and promote gender equality contributes to the overall development and progress of the nation [13]. Rwanda sets a positive example for other countries. It highlights the idea that empowering women is not only a matter of rights but also a strategic investment in the social and economic prosperity of the nation.

The absence of overt disrespect or abuse during childbirth does not guarantee the presence of RMC practices; for instance, the lack of physical abuse does not necessarily indicate the existence of compassionate and positive care behaviours [14]. RMC and mistreatment during childbirth represent opposite ends of the spectrum. Research suggests that women and newborns can experience a mix of both positive RMC practices and negative mistreatment behaviours within this range [14].

Providing RMC plays a crucial role in enhancing the quality and utilization of maternity services [3, 15, 16]. Advocating for RMC is essential for advancing the Sustainable Development Goals (SDGs), particularly in achieving the targets of eliminating preventable maternal and neonatal deaths and ending all forms of violence against women [17]. Although in 2018, WHO launched a shift towards promoting positive childbirth experiences, appreciative RMC practices have largely remained under-examined in academic literature [2], particularly in Rwanda, where very little is known about the perceptions of healthcare providers (HCPs) towards maternity care received by mothers.

Little is known regarding perceptions of HCPs regarding RMC provision in Rwanda. Ndirima published findings on perceptions of maternity services at Mibilizi District Hospital in Rwanda’s Western Province. The study involved rural women who had hospital births. The mothers commonly emphasized the need for respectful treatment in spaces which offer ample privacy, preferences regarding the gender of the birth attendant and the potential presence of their husbands during the childbirth process. Women in these remote areas make considerable efforts to access healthcare facilities with positive expectations. However, the staff shortages pose obstacles [18].

Violations against women’s dignity and respect were reported by women in labour in the health facilities of Rwanda [19]. Women were slapped, verbally berated, humiliated, reprimanded, physically assaulted, insulted, abandoned, denied having birth companions, and some were subjected to inappropriate and rough handling by healthcare providers. Many reported being shouted at for failing to follow instructions, and some were retained within hospitals due to unsettled bills [9]. In addition, women were being subjected to shouts and derogatory remarks from HCPs [19].

## Methodology

*Research Design*: This study employed an exploratory qualitative descriptive design, incorporating both Focus Group Discussions (FGD) and individual interviews as methods of data collection. The data collection approach utilized Appreciative Inquiry (AI), encouraging participants to reflect on their most positive experiences and perceptions and fostering a constructive and empowering perspective [6, 20]. This study is a part of a doctoral project aimed at gaining insights into healthcare professional’s perceptions of RMC, with the overarching goal of developing strategies to promote RMC in Rwanda. The interview guide was developed for this study and was not published elsewhere.

### Appreciative Inquiry approach

Appreciative Inquiry (AI) adopts a 5D cycle comprising the stages of Definition, Discovery, Dream, Design, and Destiny [6, 20]. This approach significantly moves away from traditional deficit-based change to a positive, strengths-oriented strategy [6, 20, 21]. AI is revolutionizing the field by presenting a proven methodology to address critical change agendas effectively [21–23]. Its focus is leveraging an organization’s ‘positive core’ strengths to develop and reshape systems, thus promoting a more efficient and enduring future [21–23]. Through AI, organizations can pinpoint positive core strengths related to the affirmative topic and take tangible steps toward achieving their objectives. Moreover, AI goes beyond being just a 5-D cycle methodology, ushering in deeper, more meaningful, and sustainable change at the organization’s heart [21–23]. In this study, HCPs expressed their thoughts on mothers’ reports from the previous phase, corresponding with AI’s dream and design stage.

*DREAM phase*: The Dream phase involves envisioning a positive future, building upon the qualities uncovered in the Discovery phase [6, 20, 21]. It stimulates conversations about desirable outcomes, prompting the question, ”What would ideal success (for RMC) entail?” Participants explore new possibilities for the future [6, 20].AI anchors future visions in the positive aspects of the past, drawing inspiration from remarkable moments in the organization’s history [6, 20, 21]. The main objectives include fostering dialogue among participants by sharing positive narratives and identifying common themes without criticism [6, 20]. The Dream phase encourages stakeholders to engage in discussions and imagine a better organization and world, stimulating the status quo to generate synergy and enthusiasm [6, 20–23].

This stage of research entails leveraging the insights gathered from the discovery phase (based on mothers’ feedback) to formulate a series of aspiration statements that inform the design of future actions [6, 20, 21]. The Dream phase visualizes a favourable future, drawing upon the qualities uncovered in the preceding Discovery phase, which documented RMC experiences reported by mothers [6, 20]. The researchers investigated health care providers’ viewpoints (based on what was reported by mothers) regarding maintaining RMC initiatives through various imaginative exercises and innovative methods. The HCPs were prompted to envision and conceptualize the future of RMC. During interviews and FGDs, the following questions were posed:

*The mother appreciates receiving compassionate care. What do you think can be done to achieve this?*

*The mother values autonomy/independence in the care received. What measures do you think can work to achieve this?*

*The mother values efficient care. What steps can be taken to achieve this? Additionally, how do you propose dignified care and respect be sustained?*

*How would you ensure the maintenance of privacy and confidentiality for women in labour?*

*Mothers prefer giving birth via normal delivery. How can these preferences be honoured?*

*Mothers desire to have a healthy baby. How can this goal be achieved?*

*Mothers prefer to stay in a clean and tidy environment. How can this preference be accommodated?*

*Mothers perceive that RMC is associated with the love of individuals and a passion for the profession. How do you propose these aspects be sustained?*

*Design*: Generate options for implementation and co-create plans by focusing on “What would constitute the ideal?” [6, 20]. The Design phase underscores the utilization of past successes to devise actionable steps towards the vision outlined in the Dream phase [6, 20]. Its primary objective is to pinpoint specific actions that align with the novel possibilities envisioned during the dream phase [6, 20, 21]. Participants work collectively to generate and commit to actions that translate aspiration statements into tangible outcomes. [6, 20, 21]. The design phase delineates the strategic direction, incorporating a vision of sustainability and a compelling statement of strategic intent [6, 20, 21]. It plays a pivotal role in sustaining positive change and realizing the organization’s fullest potential, as exemplars from past successes shape future projections [6, 20]. During extensive dialogues, stakeholders delineate the necessary processes to uphold the new system, ensuring it aligns with the desired outcomes of the inquiry [6, 20–23].

Examples of questions posed include the following: *The community holds various perspectives, whether positive or negative, regarding the care provided to women during labour and childbirth. How can trust be restored within the community?*

*What interventions or actions by healthcare providers would exemplify RMC?*

The HCPs suggested maintaining and enhancing the most effective RMC practices. In this study, participants suggested strategies, protocols, and systems. They established partnerships that ultimately fostered and bolstered positive changes in RMC among themselves as HCPs. They developed inspiring intentions and precise design statements. Consequently, the detailed visions/strategies derived from the previous phase’s findings and mothers’ perceptions enabled the HCPs to formulate their plans to address such.

*Destiny*: (We did not reach this stage). The Destiny phase represents the culmination of discovery, dream, and design, establishing an “appreciative learning culture.” [6, 20]. It is the implementation stage, bringing the envisioned new future into reality and fostering a collective sense of purpose [6, 20]. This phase involves ongoing learning, adaptation, and innovation, aligning interactions with shared ideals to ensure long-term success [6, 20–23].

*Study Setting*: The research was conducted at five hospitals located in the Eastern province of Rwanda. The Eastern province was selected because it is the second province with the highest birth rate based on the latest report from Rwanda 2019–2020 Demographic and Health Survey [24]. We have chosen these hospitals because they admit and care for both normal and complicated deliveries.

*Population and the sampling method:* The population consisted of healthcare providers (HCPs) working in maternity services in the Eastern province of Rwanda and included medical doctors, midwives, and nurses. We purposefully recruited healthcare professionals (HCPs) from each of the five selected hospitals, ensuring they had worked there for at least one year. The day before data collection commenced, the Principal Investigator reached out to the Nursing Director and maternity unit supervisors (matrons) at each hospital to discuss the data collection plan. On the day of data collection, the researcher invited HCPs to participate in the study. We considered both off-duty and on-duty HCPs eligible for participation. Recognizing the demanding nature of maternity services and the busy schedules of staff, we remained flexible with timing for those on duty, conducting interviews during their break times. We provided thorough explanations of our study to all participants, and those who chose to take part signed a consent form.

We conducted ten in-depth interviews involving five medical practitioners and five matrons (refer to Table 1). Additionally, five Focus Group Discussions were held, with the participation of 40 midwives and nurses working in maternity care (refer to Table 2). The investigation took place in May 2023. In general, participants were asked about their opinions on the findings reported by mothers in a previous study (Muhayimana A, Kearns IJ, Gishoma D, Tengera O, Uhawenimana TC: Experiences and perceptions of respectful maternity care during childbirth in health facilities of Eastern province of Rwanda: An Appreciative Inquiry, unpublished).

**Table 1:** Socio-demographic data from individual interviews *n* = 10

***n***=10			
Variables	Categories	Frequency	Percentage%
**Age**	25–30	4	40
	31–35	3	30
	36 and above	3	30
**Sex**	Female	2	20
	Male	8	80
**Profession**	Medical doctor	5	50
	Midwife	5	50
**Work Experience in maternity**	1–5 years	6	60
	6–10 years	4	40

**Table 2:** Socio-demographic data from Focus Groups *n* = 40

***n=40***			
Variables	Categories	Frequency	Percentage%
**Age**	25–30	11	27.5
	31–35	19	47.5
	36 and above	10	25
**Sex**	Female	30	75
	Male	10	25
	Midwife	29	72.5
	Nurses	11	27.5
**Work Experience in maternity**	1–5 years	20	50
	6–10 years	15	37.5
	11 years and above	5	12.5

*Data collection*: The interview was conducted in either Kinyarwanda or English, depending on the participant’s preferred language. The interviewer was a Faculty member from the University of Rwanda, in Kigali and the study was conducted in a province where the interviewer and interviewees were not known to each other, hence, no power relationship existed.

The interviews and FGDs were conducted in a designated private room in the hospital. The participants were assured about the total confidentiality of their information throughout the whole process. Participants in IDIs was assured that their names and other identifying information will not be recorded anywhere and that no individual participant will be identified in any report or publication arising out of this study. However, in the FGDs, because of the group nature of a Focus Group, we kindly asked them to treat the discussions as confidential.

Demographic details were collected using a socio-demographic questionnaire. We used a semi-structured interview guide with open-ended questions (Note the questions in the appreciative inquiry approach section). In-depth information was elicited through probing questions. Participants were informed that mothers value compassionate care, and we asked for their suggestions on providing care free from harm and ill-treatment, as well as ways to demonstrate empathy. The interviewer emphasized that mothers appreciated autonomy and independence and asked the participants how to achieve this. The interviewer asked the participants how they could grant mothers decision-making liberty, allowing mothers to express their choices and preferences and providing them with relevant information. Additionally, participants were informed that mothers value-efficient care, and the interviewer sought their input on achieving this while sustaining dignified care, respect, and maintaining privacy and confidentiality.

Furthermore, HCPs were informed that mothers expressed a desire for a normal delivery, getting a healthy baby, and staying in a clean and tidy environment. The interview inquired about actions that can be taken to uphold these preferences. HCPs were also informed that mothers typically perceive HCPs who provide RMC as those who have a love for people and a passion for the profession. The interviewer asked how these aspects can be sustained. HCPs were then reminded that the community holds varying perspectives, both positive and negative, regarding the care women receive during labour and the birth process, and they were asked to propose ways to regain the community’s trust, along with interventions or actions from HCPs that would demonstrate RMC. Furthermore, HCPs were queried about the methods they view as respectful towards a woman in labour, from a healthcare provider’s perspective, from hospital management, and within health facilities.

Following the conclusion of each interview, the researcher stressed the significance of subsequent communication via telephone calls to review the study findings and confirm their precise representation of participants’ perceptions. Throughout the discussions, descriptive field notes were documented during the interviews and Focus groups, and any fresh insights offered by participants were integrated into the analyzed data.

*Data Analysis*: After each interview, an experienced transcriber immediately, manually transcribed the audio recordings word for word. Guided by the principal investigator, a research assistant translated these transcripts into English, simplifying the process and maintaining data integrity. These translations were then reverse-translated into Kinyarwanda to verify accuracy. The research employed an inductive thematic analysis approach, utilizing pertinent quotes to convey the findings. To deeply engage with the data, the researcher extensively reviewed the transcripts. The Nvivo 12 software played a crucial role in organizing the transcribed data.

The research team initiated the process by carefully listening to the recorded audio, ensuring the completeness and integrity of the data through cross-referencing with transcriptions. To gain a thorough understanding of the content, the team meticulously examined the transcripts [25]. They utilized line-by-line coding to identify initial codes, which were then evaluated to establish categories based on similarities and differences in participants’ responses. This process involved an iterative approach, with the team continually revisiting and refining the categories while cross-referencing with the original transcripts to ensure they accurately captured participants’ genuine experiences and perspectives [25]. This rigorous analysis led to the identification of primary themes. Participants were consulted through a process known as member checking to validate themes [26], ensuring that the themes resonated with their perceptions. Subsequently, an inductive thematic analysis was conducted, complemented by relevant quotes to present the findings comprehensively [27].

### Trustworthiness measure

To ensure the trustworthiness of the research findings in this study, the researcher adhered to the evaluative criteria for rigour in qualitative research. These criteria, as outlined by Lincoln and Guba [28] and Grove and Burns [26], encompass credibility, dependability, confirmability, and transferability. Credibility was ensured by deliberately selecting healthcare providers who met eligibility criteria, using open-ended questions in interviews to elicit detailed responses, and incorporating peer debriefing and member checking during data collection and analysis. Sharing transcripts for accuracy confirmation further enhanced credibility. Dependability was maintained by meticulously documenting decisions from research initiation to data analysis, creating a comprehensive audit trail. Data stability was also upheld through timely and consistent collection under similar conditions within a specific, relatively short period. Confirmability was maintained by relying solely on participant narratives for analysis, avoiding the imposition of the researcher’s assumptions [29] [26]. Transferability was enhanced by providing a detailed description of the study’s setting, sample, and investigated phenomenon using a well-structured interview guide.

## Results

The majority (70%) of participants who took part in the interview were aged between 25 and 35. Among them, 80% were male, and 60% had experience of up to only five years. (Table 1)*.*

About half of the participants in the FGDs ranged within the age of 31 to 35 (49.5%). The majority of participants were midwives (72.5%), and approximately half of them had accumulated one to five years of experience working in maternity services.(Table 2)*.*

During thematic analysis, four themes and sub-themes emerged from the data:

1. Women-centered care with Compassionate care, Privacy and confidentiality maintenance, Information provision and Liberty in decision making, Effective communication, Family involvement, Cleanliness of environment, and Equality care.
2. Professionalism compliance with Motivated staff, Teamwork, Continuous development, Quality work provision, and Community trust.
3. RMC encounters*.*
4. RMC sustenance.

### Women centered-care

#### Compassionate care

Healthcare providers emphasized the importance of showing compassion to mothers. They stressed the need to put themselves in the client’s shoes, treat the mother with empathy, reflect on ethical considerations, ensure her confidentiality, care for her with a good heart, make the mother feel relaxed, and show love and hope. They added that truly compassionate service would be achieved if a doctor treated every mother like their own mother, wife, or sister. HCP reported that performing episiotomy without anaesthesia no longer exists.

*“We need to have a heart of love and patience. In maternity, there is hard work, but we should have the heart to love people. So first we should strengthen love in ourselves and then have the gift of patience … What we should do for the mothers to show them compassion first is to put ourselves in their position because if we put ourselves in their place.” FGD1.*

HCPs reported that mothers should understand that labour pain is natural and normal. HCPs support labour by reassuring the mothers, providing back massages, encouraging them to ambulate, and ensuring emotional well-being by offering comforting words. Service providers reported having insufficient knowledge about labour painkiller medications.

*“An area where we have little knowledge is pain relief for the mother in labour. Pain management is not appropriately done, but we teach them with the help of the Bible”.* FGD 5.

#### Privacy and confidentiality maintenance

HCPs emphasized the criticality of maintaining privacy and confidentiality in healthcare settings. They noted that while curtains between beds are used to uphold privacy, the use of rooms is recommended as curtains may not always ensure confidentiality. HCPs also highlighted the importance of maintaining confidentiality during key processes such as handovers, labelling laboratory samples, and medical rounds, where they employ medical terms, signs, and identifiers. In our study sites, participants shared that they have transitioned from using printed files to utilizing technology (such as open clinic) for client information storage, with each individual having a unique login password. However, concerns were raised about preventing providers from accessing maternity information from other services. In one study site, plans were underway to construct maternity rooms to enable birth companionship.

*“Mainly, we care about the information we give to the mothers; their information is confidential, and we care about privacy”.* IDI, matron, female, 34 years.

#### Information provision and liberty in decision making

Midwives reported that a mother has the right to request information and receive it, and she also has the right to make choices and express preferences. Participants reported that the rights and responsibilities of the clients are written on the hospital wall. Some information for mother and neonatal care is being provided. Some participants mentioned that during the reception, customer care services welcomed the mothers and allowed them to contact the representative of customer care services at any time during their stay in the hospital. Additionally, before being admitted, mothers are required to sign a consent form. Participants recommended that women’s rights should be posted in a visible location for mothers to read.

*“There is a customer care representative of the hospital; her photo and number are available; they call her if they cannot find the matron. If a person has a problem and brings it to customer care, it is solved quickly*”. IDI, matron, female, 33 years.

Participants emphasized that mothers have the right to make their own decisions before receiving care. A mother may not know about her health; therefore, HCPs should first provide all information to the mother by explaining the pros and cons, allowing her to make a decision, and then sign the consent.

*“The care is central to women, not to us. She is the one who is going into labour; she is the one who is going to bear any procedure that we are going to perform on her, so she has the right to refuse the treatment.”* IDI, doctor, male, 30 years.

Participants reported that a mother has the right to choose the treatment; for example, she has the right to opt for a cesarean section without medical indication. The decision-making process for a mother’s treatment should be done as quickly as possible so that HCPs can take action without unnecessary delays.

#### Effective communication

A suggestion box is available for clients to share their ideas in order to promote collaboration and mutual respect among clients, service providers, and hospital management. This feedback is reviewed and thoughtfully considered, empowering clients and ensuring they feel heard. Additionally, some hospitals offer a complaint book for clients to submit grievances, ensuring that their concerns are taken seriously and appropriate measures are taken in cases of disrespect.

There is a way to handle incidents of disrespect.

*“When there is an incidence of disrespect, we have a quality book; whoever is found to have happened is approached and discussed, and we can register her in the book and resolve an issue with the victim. If this happens for the second time, the case is presented to the higher authorities for punishment:” IDI, matron, female,33 years*.

To handle disrespect issues, HCPs from one hospital reported that they manage it at their level or they refer to mental health services.

*“If a client is physically or psychologically harmed by a healthcare provider, the first is to prevent the spreading of information. In our measures, we have a team leader who must handle the problem and approach the family to find a solution. If it is beyond our control, we can use the mental health service to help us.” *IDI, matron, male,27 years.

To enhance client-provider communication, providers must explain the labour process to the clients, giving them ample time to ask questions freely. This open dialogue provides clients with the necessary information and makes them feel respected and cared for. Listening to their concerns and providing feedback during this process helps to establish trust, fostering a positive healthcare experience.

*“If you have confidence in explaining to the mother how things are going, and when you explain directly to her, she immediately trusts you”*. IDI. Doctor, male, 37 years.

Participants advised HCPs first to introduce themselves to the mother. Taking time to read the client’s file and talking with the client helps avoid harm. Explaining everything to the mother, including information on the baby’s care, lab tests, and results, is crucial. Participants emphasized the importance of consented care, stating that service providers should explain everything they do and its significance, ensuring that mothers also understand. Providers should also clarify what is and is not allowed in the hospital, explaining their rights and responsibilities. They added that sometimes it is challenging to find time to provide all explanations to the mother.

*“First, we respect her rights. Respecting the right of the mother is to listen to her, to explain to her what she wants to know.”* FGD1.

#### Family involvement

HCPs argue that a mother has the right to involve her family in her care process, and service providers help the mother to achieve that. Midwives used to involve a birth companion to assist in the mother’s care. However, the infrastructure hinders the birth companion from staying with the mother during the second stage of labour and is a significant challenge.

*“The mother has the right to involve her family in the treatment when she wants so that the male companion can come if she asks us, but when she does not ask, we have to treat her according to her rights.” *FGD 3.

#### Cleanness

Service providers argue that they encourage mothers to maintain personal hygiene. For infection prevention, HCPs used to request mothers to wear diapers during labour to prevent the spreading of vaginal leakage. However, some mothers with traditional beliefs resist wearing those diapers. Providers ensure the cleanliness of beds and rooms and encourage mothers to maintain personal cleanliness as well. Cleanliness in the service is maintained, and some cleaners follow waste management measures specifically for the maternity ward. There is a daily cleaning schedule, as well as total cleaning. They stated that cleanliness should be a cultural norm.

*“It is crucial to keep the hospital clean. To keep it going, everyone in the service is responsible for the mother’s hygiene, not just the nurse”*. IDI, Doctor, male, 37 years.

Participants recommend providing clean water and soap for clients, along with outlining plans for bathing and bedmaking. They also suggest requesting mothers to come with their hygienic supplies.

#### Equality care

Service providers echoed that they provide equal care to all women, accepting them as they are without considering their socio-economic status. However, they reported that educated mothers understand things more simply and quickly than others. Participants questioned why previous findings on quantitative research conducted on mothers reported that mothers who delivered by caesarean section reported being more respected than those with a normal delivery. They pointed out that a team performs a caesarean section, whereas in a normal delivery, the mother is cared for by one or two persons. Thus, a mother may simply be biased, thinking that being cared for by a team is a sign of respect.

*“For mothers who delivered by caesarian section, it is not a procedure that is performed by one healthcare provider. There are many anesthesiologists and obstetricians… there are many. In the operating room, you find 4 to 5 people around you; this leads the mother to think she is really cared for. However, for vaginal delivery, there is only one person. ”*FGD 2.

In addition, they reported that in a cesarean section, the mother is given pain medication before, during, and after the procedure. In contrast, during vaginal delivery, the mother may struggle to cope with labour pain, leading to feelings of giving up, and sometimes mothers are mistreated in an effort to save the baby.

### Professionalism compliance

HCPs reported that their profession is a vocation and not like other professions. The love for the profession and respect for the oath are considered very important. Midwives reported that they relate their practices to what they learned in school. Furthermore, they learn from their experiences and avoid repeating past incidents. HCPs reported conducting client interviews and sharing client feedback with the staff. In meetings, the unit manager shares what is appreciated and critiques themselves. Employees know they should provide good services and be safe and calm in difficult situations. There is a culture of prioritization involving triage, starting with emergency cases. The challenge is a large number of clients and an insufficient workforce. There is a culture of listening to the client’s complaints and making incident reports if they occur.

*“We have established a culture among the staff that if our clients complain, you should not tell them that I cannot help them, but you should listen to them, help them and give them advice”* IDI, matron, 34 years**.**

#### Motivated staff

The matron reported that in order to motivate the team, they have established solidarity and social activities among the maternity team. Midwives organize and attend social events of their colleagues, such as wedding ceremonies and birth events, and even visit colleagues during challenging moments. They added that what keeps them in their career is the feeling that they are saving people, and when mothers are happy, HCPs also become happy (a positive cycle). Participants suggested ways to motivate the staff, such as providing tea or even food to decrease work stress and burnout, as well as rewarding and appreciating the best employees in public.

*“When an employee does something good, you should praise her. Appreciating her is one thing; do not give her money because she is being paid, but showing her that you value what she did; this is also a motivation”*. IDI, matron, 33 years.

#### Teamwork

The participants reported that teamwork is key, and everyone in a team should be aware of their limitations and ask for help so that the mothers can achieve better outcomes and happiness. The task allocation schedule exercise is flexible, considering every staff member’s issues without compromising the work. They advised if the health personnel don’t feel well, it is better to communicate their feelings to their colleagues, and the staff should be attentive to those feelings to prevent any negative mood from affecting the mothers.

*“There are times when a midwife comes with problems. We should know who has a problem so that it can disturb the client’s feelings, and we assign her to a place where she will not meet people”* FGD 4.

#### Continuous development

Some midwives have gotten RMC training. One matron requested continuous professional courses at the hospital or school. The training can be organized by the hospital or the Ministry of Health. Midwives need the opportunity to receive full scholarships to upgrade their educational level.

*“Here we have midwives who have been around for more than 15 years, and the healthcare system is being updated. Therefore, for mothers to receive quality care, it is essential to increase their knowledge.”* IDI, matron, male,27 years.

#### Quality work provision

Participants reported that accountability is needed to sustain the quality of care. HCPs emphasized their pride in getting a healthy baby and a healthy mother. Planning for pregnancy was highlighted as a key to a successful outcome. Participants appreciated that neonatology rooms are available. Participants stressed the importance of avoiding carelessness, implementing strict labour monitoring, following the partograph, and complying with accreditation guidelines. Hard copies of protocols are available, and HCPs are able to check online for national guidelines when they encounter challenges. These hospitals are in the process of accreditation to improve the quality of services. Some HCPs reported that they have regular meetings and audits to monitor and evaluate the standards. Employee evaluation has a significant impact on RMC provision. Every staff member receives a bonus based on competence-based performance (PBF), which motivates them to make an effort to work better.

In hospitals, a survey on customer satisfaction is usually conducted, and recommendations based on the feedback are implemented. The project on quality improvement conducts evaluations every month. Protocols from the Ministry of Health are provided to every staff member.

*“When a mother gives birth to a healthy baby, and all of them are healthy, it is our pride as midwives and the pride of the country and the community in general”. *FGD 3.

In the past, some hospital buildings did not have immediate access to macadamized roads. The HCPs appreciate the construction of a macadamized road to their hospital.

*“The road to this hospital was damaged, the cars used to get there were going to stack, and you found mothers complaining about it, but now the road is macadamized and enjoyable, and mothers feel respected.” *FGD1.

### Community trust

The participants believe that when they provide friendly service, mothers will repeatedly share their positive experiences within the community, thereby restoring trust in the community.

*“To gain community trust, we need a good outcome. Whenever you treat a woman with empathy, she will be the one to preach to others in the whole village. The one you treat well is the one who is going to change the population’s mind”*. IDI, doctor, male, 30 years.

*“We have a program called the patient’s voice, as well as visit the community and listen to feedback and recommendations”.* IDI, matron, male, 32 years.

### RMC encounters

Labour pain medications are not yet available at all study sites. At the national level, labour pain medication protocols are available but only followed in tertiary hospitals in the city. However, doctors in our study sites may not have the necessary skills to administer subdural anaesthesia. In the postpartum period, minor painkillers like paracetamol and tramadol are administered.

*“There are labour pain management protocols that have been developed at the national level, but the nature of the hospital does not allow doctors to give labour painkiller drugs. Still, in our hospitals in Rwanda, we do not have enough doctors to care for the mother who has been given subdural anaesthesia.”* IDI, doctor, male, 35 years.

Participants reported that mothers are taking traditional medications, which can lead to maternal and neonatal adverse outcomes.

*“Although it is not scientifically proven, we do not know what key ingredients are included in traditional medicines. Sometimes you find that mothers are saying they help them, but they can also harm the condition of the baby in the womb.” *IDI, doctor, male, 35 years.

Among reported hindrances to RMC are insufficient labour wards; sometimes, birth companions fear seeing mothers screaming and do not understand it. Most maternity services have insufficient doctors and midwives, overloaded clients, insufficient equipment like one cardiotocography (CTG) machine per 20 clients, and a lack of private rooms. In addition, available rooms are built in close proximity so that one person can hear the other, which hinders confidentiality, privacy, and staying with a birth companion. Insufficient beds where 2 or 3 mothers share one bed have been reported. One matron reported that there is a staffing plan that shows the workload, and they advocate to the Ministry of Health to increase the number of midwives.

Service providers argue that it is mostly difficult to explain to the mothers and meet their level of understanding because many mothers are uneducated. Providers from one hospital reported that obtaining clean water in the hospital is a problem, but the issue has been reported to the hospital management to find a solution. Participants argue that the service can be good, but client satisfaction depends on someone’s perceptions and personality.

### RMC sustenance

To sustain RMC, participants emphasized that an empathetic approach towards mothers should be a cultural norm. They added that having self-confidence, regular supervision (with the clinical director overseeing technical staff and correcting errors), being reminded of fundamental human rights principles, and avoiding rushing in care provision to avoid being busy on computers. Regular quality improvement meetings and RMC refresher training should reinforce the monitoring system. Workload adjustment, educating mothers on their rights at the hospital entrance. Equipment should be requested on time; collaboration with maintenance services is crucial.

Health education should be conducted during antenatal care and at the community level to discourage the use of traditional medicine. Close monitoring during labour is essential, and increasing the number of workers and implementing shifts is recommended. The participants advocated for receiving motivation, such as salary increases or bonuses, and provided refreshments like tea breaks, which were encouraged. There is a suggestion to increase the capacity of customer care services to be available 24/7. Participants also recommend organizing study trips to hospitals with high standards.

*“I feel what could be done; there is something called a school trip; I felt that we can learn from another hospital by visiting the model place …We can learn how their maternity, and how they maintain the privacy of their patients…. We should learn from others to see how they do it, as said in Kinyarwanda’s proverb, “A bird that does not fly does not know where the corn is ripe”. *FGD 4.

The HCPs argue that to enable mothers to have a normal delivery, mothers are encouraged to attend antenatal consultations, perform physical exercises, and attend the hospital early. In addition, participants emphasized that the mother should be called by name.

*“If you call the mother by name when she sees you call her by her name; it makes her feel comfortable”*. FGD 1.

Participants reported that they should be diligent and provide better service for better outcomes to gain community trust. The community should be informed about the services typically offered and those beyond their capacity. Additionally, HCPs should focus on positive changes, maintain positive aspects, learn from others, and constantly self-evaluate. HCPs should improve patient education and receive mothers well, as the client’s first impression can play a role in regaining community trust. HCPs advised the hospital management to determine the community’s perceptions of hospital services.

Many participants suggest increasing the staff, especially midwives, to perform normal deliveries, train newcomers, and improve the buildings, notably phasing out the use of curtains. Some participants suggested using soundproofing to maintain the confidentiality of mothers. They recommended using rooms with soundproofing instead of curtains to ensure patient information cannot be overheard by other patients.

They recommended monitoring the mother adequately and teaching her how to behave, position, and breathe during labour. It is suggested that enough equipment like CTG machines be procured based on the number of clients, adequate numbers of beds should be ensured, and cleanliness in the hospital should be supervised. Participants emphasize working with a heart of love, reinforcing awareness of women’s rights on the radio or other platforms, and conducting RMC sensitization in the community. They advise to avoid retaining mothers in the hospital when they fail to clear the medical bill.

## Discussion

In the context of this paper, the participants’ perceptions highlight the importance of ensuring women-centred care, with compassion, privacy, confidentiality, the right to information, autonomy, effective communication, holistic care with family involvement, personal hygiene, and equal care. Women-centred care aligns with the experience of care dimensions established by the WHO in 2018 and the quality of care framework for maternal and newborn health [7]. Shakibazadeh et al. [30] identified twelve domains of RMC, one of which emphasizes providing woman-centered care.

A study employing Appreciative Inquiry (AI) conducted in Tanzania and Malawi reported that compassionate healthcare provider-woman interactions involve a warm reception, polite language, and timely and pertinent care, as well as the exchange of relevant information [17]. The utilization of woman-centered care leads to positive childbirth experiences and positive outcomes for both mothers and newborns [31]. Moreover, to achieve a significant reduction in maternal mortality, there should be a strong focus on making services truly woman-centred [32]. Surprisingly, in a study conducted in Ghana, midwife students attempted to justify mistreatment. The midwife students argue that if a mother in labour is uncooperative, in order to avoid adverse outcomes, the midwives have a reason to beat her a bit to ensure she complies with instructions [33]. Studies highlighted the need to raise awareness among care providers about RMC standards by enhancing client-provider communication, monitoring practices, and strengthening accountability mechanisms for health workers [31, 34].

Participants also revealed that compliance with their profession is key to achieving RMC. Professional compliance becomes possible when staff work as a team. Studies argue that teamwork among all HCPs is associated with improvements in respectful care [5, 32]. The positive attitude of HCPs is crucial, even in attracting mothers to utilize health facility childbirth [10, 17]. Supportive leadership is key to successful RMC practices [17, 35–37].

A conducive environment with the necessary equipment and adequate physical setting leads to positive childbirth experiences [17, 38, 39]. Improving infrastructure may be perceived as challenging to implement and could take time. However, HCPs can work with existing resources, such as using partitions and curtains, to maintain the privacy of mothers [17]. In our settings, though the rights and responsibilities of clients are displayed on the hospital wall, these rights are general patient rights and are not specific to women and newborns. There is a need to post the RMC charter on the maternity wall as a strategy for creating awareness of RMC among service users.

In some hospitals from the study sites, there is a culture of systematically obliging every woman in labour to wear a diaper for individual hygiene and to prevent attracting or spreading infection. However, some women reported resisting, fearing that their babies could die inside. It is crucial to explain to the mothers everything in order to alleviate the mothers’ anxiety, as recommended by WHO [40]. In this particular case it is important to reassure the mothers that there is no harm in wearing a diaper before giving birth. Traditionally, Rwandan women do not even put on underwear during labour because they believe it can delay the descent of the baby, leading to prolonged labour or the baby being asphyxiated inside.

Participants recommended improving services and explaining available services based on the hospital level to build community trust. An interventional study in Kenya suggested using open maternity days to engage the community in maternity services [10]. To foster a sense of belonging and ownership, it is essential to involve the Rwandan community in maternity service activities during community work, such as building and cleaning health facilities. The community should also be educated about the rights of women and newborns.

Though the purpose of this study was to explore the positive aspects of what can be done, the participants also narrated the challenges that hinder the RMC provision. They reported several issues affecting RMC, including insufficient labour wards, sharing of beds, inadequate birth companionship, non-use of labour painkiller medicines in all health facilities, staff shortages, client overload, and a lack of equipment. The absence of private rooms hampers confidentiality, privacy, and the ability to stay with a birth companion [15]. Challenges in delivering RMC included logistical difficulties in ensuring privacy, lack of mobility during labour, high workload, absence of alternative birthing positions, negative attitudes from certain midwives, and language barriers [14, 15].

Supportive leadership, which oversees the staff, supervision, and mentorship, was also recommended. Evidence reported that the lack of RMC is not only an individual issue [10] [38, 41]. According to Kujawski et al. [38], RMC is not attainable through a straightforward single technical solution. Instead, eliminating disrespect and abuse necessitates individual behaviour change, organizational and systemic adjustments, and, ultimately, a more profound societal transformation [38]. Therefore, achieving RMC is a process that requires evidence-based strategies to achieve the deliverables and commitments that healthcare practitioners are called on and declared upon [42]. These strategies include the establishment of national laws and policy standards for RMC, adherence to human rights and ethical principles, and the presence of effective governance and leadership [17, 42].

Contributors to RMC encompass having an ample number of HCPs trained in RMC, ensuring the availability of sufficient drugs, equipment, and technology, maintaining a functional information system, and securing financing, such as health insurance [42]. It is essential to have well-equipped health facilities with adequate infrastructure, delivering care with accountability and supervision [42]. Individual and community involvement is crucial, with mothers empowered to autonomously demand RMC and make the community aware of RMC principles. Furthermore, a complaints redress mechanism should be implemented [10, 41]. It was proved that multiple interventions are more effective in improving RMC compared to a single intervention [15, 35–37, 42].

Respect is fundamental to Rwandan culture, influencing various traditional social interactions. Integrating this culture of respect into the journey of childbirth is essential to enhance the contributions of Rwandan women and mothers to the nation-building process [13]. RMC aligns with Rwanda’s culture of respecting everyone, especially pregnant women. Rwanda is fostering a culture that values and invests in the well-being of its women and mothers. However, it should be noted that predictors of positive or negative RMC experiences can differ across countries, settings, and cultures.

The methodology we used in this study is appropriate to RMC; appreciative inquiry promotes positive thinking and uplifts and sustains existing best practices rather than struggling to repair harm [17]. The AI method brings suitable solutions for countries with resource-constrained health settings like Rwanda because it fosters a culture of maintaining positive traits, working with what is available in the field, and appreciating what has been achieved instead of perpetuating blame [6, 17].

Rwanda has made progress towards universal health coverage. Presently, 91% of Rwandans have medical health insurance [43], resulting in a significant decrease in post-childbirth hospital detentions due to unpaid bills. RMC is now integrated into Basic Emergency Obstetric and Newborn Care (BEmONC), with healthcare providers receiving RMC training and mentorship through programs such as the Maternal and Child Survival Program [44] and the More Happy Birthdays project [45]. These initiatives are conducted in collaboration with the Rwanda Association of Midwives and the Ministry of Health, but efforts to enhance RMC in Rwanda are still less documented [46].

## Conclusion and recommendations

The pursuit of a RMC high standard is an ongoing journey, and some steps have been taken to improve RMC in Rwanda. Therefore, in the ongoing process of achieving RMC, it is possible to enhance the childbirth experience by working with existing resources, continuously improving over time, and sustaining the achievements until reaching the RMC standard.

In this study, the recommended actions to sustain RMC include promoting women-centred care, improving healthcare providers’ attitudes, abiding by the profession, building community trust, ensuring a conducive environment in health facilities, and leadership should be actively involved and provide support.

## Strengths and limitations

One strength of this study is the utilization of the Appreciative Inquiry approach, which provided a positive way forward by shifting from problem-focused to solution-seeking perspectives. AI is particularly suitable for RMC because it focuses on the best aspects, is less threatening for HCPs, and can potentially reveal creative solutions and ideas that may not emerge solely from addressing disrespect and abuse. AI offers the advantages of genuine commitment, creating energy and motivation. AI is engaging, powerful, inspiring, and focuses on the positive aspects of situations [6, 20]. The recommendations yielded from this study are applied to low-resource health settings, and improvement of RMC working with the currently available resources are envisaged. This study’s limitations stem from exclusive reliance on participants’ self-reported experiences and the viewpoints of the individual participants. AI may not be suitable for some circumstances, especially when immediate action is needed [47]. Transitioning to a strength-based approach requires persistence, practice, and patience [47].

### Supplementary Information

Supplementary Material 1.

Supplementary Material 2.

## Data Availability

Data is provided within the manuscript supplementary information files ” The datasets generated and analyzed during the current study are available in the Supplementary Data1_ Excel output from Invivo 12 analysis of health care providers’ interviews and focus groups repository.
