# Supplementary material for: Healthcare providers’ perspectives on sustaining respectful maternity care appreciated by mothers in five hospitals of Rwanda
Source: BMC Nurs. 2024 Jun 28;23:442. doi: 10.1186/s12912-024-02017-5 (PMC11212382; doi:10.1186/s12912-024-02017-5)
Supplement: Supplementary file 2 — Supplementary Material 2. [file 12912_2024_2017_MOESM2_ESM.pdf]

Guide for Interview and Focus groups

1. To explore Health Care Providers **views of RMC** as reported by the women from Phase 1 of the study using **FGDs and Key Informant Interviews**

IDIs are for maternity unity managers and doctors working in maternity services

FGD are for nurses and midwives working in maternity services

---

Q1. The mother appreciates to receive compassionate care. Tell me what you think can be done to achieve this?

Probe: What do you propose to provide as care that are free from harm and ill treatment?

Probe: What do you think can be done to show empathy to the mothers during labour?

Q2. The mother appreciates to have autonomy/independence in care received. What do you think can work to achieve this?

Probe: What do you propose to provide liberty to mother in decision making?

Probe: What can work to allow the mother to make her own choices and preference during labour?

Probe: What do you think can be done to sustain informing mother on her own health information and of the newborn?

Q3. The mother appreciates to get efficient care. What do you think can work to achieve this?

Q4. How do you think dignified care and respect can be sustained?

Q5. How would you maintain the aspects of privacy and confidentiality for women in Labour?

Q6 Mothers desire to give birth by normal delivery.

Probe: How can these aspects be upheld?

Q7 Mothers desire to have a healthy baby.

Probe: How can these aspects be upheld?

Q8 Mothers desire to stay in a clean and tidy environment.

Probe: How can these aspects be upheld?

Q9. Mothers perceive that RMC is associated with love of persons and love for the profession.

Probe: How can these aspects be sustained?

Q10 The Community has different perspectives (either good or bad) on the care that women, receive during labour and the birth process.

Probe: How can you propose to gain the community's trust back?

Q11. What interventions or actions from the HCP would display RMC?

Probe: What approaches to the woman in labour do you consider as respectful by a health care provider?

Probe: What approaches to the woman in labour do you consider as respectful by hospital management?

Probe: What approaches to the woman in labour do you consider as respectful by health facilities?
